# Src inhibition modulates AMBRA1‐mediated mitophagy to counteract endothelial‐to‐mesenchymal transition in renal allograft fibrosis

**DOI:** 10.1111/cpr.13699

**Published:** 2024-06-29

**Authors:** Zeping Gui, Xuzhong Liu, Zhen Xu, Dengyuan Feng, Zhou Hang, Ming Zheng, Hao Chen, Shuang Fei, Li Sun, Jun Tao, Zhijian Han, Xiaobin Ju, Min Gu, Ruoyun Tan, Zijie Wang

**Affiliations:** ^1^ Department of Urology The Second Affiliated Hospital with Nanjing Medical University Nanjing China; ^2^ Department of Urology The First Affiliated Hospital with Nanjing Medical University Nanjing China; ^3^ Department of Urology Huai'an First People's Hospital, Nanjing Medical University Huai'an China; ^4^ Department of Urology Affiliated Clinical College of Xuzhou Medical University Huai'an China; ^5^ Department of Urology The Affiliated Taizhou People's Hospital of Nanjing Medical University Taizhou China

## Abstract

Chronic allograft dysfunction (CAD) poses a significant challenge in kidney transplantation, with renal vascular endothelial‐to‐mesenchymal transition (EndMT) playing a vital role. While renal vascular EndMT has been verified as an important contributing factor to renal allograft interstitial fibrosis/tubular atrophy in CAD patients, its underlying mechanisms remain obscure. Currently, Src activation is closely linked to organ fibrosis development. Single‐cell transcriptomic analysis in clinical patients revealed that Src is a potential pivotal mediator in CAD progression. Our findings revealed a significant upregulation of Src which closely associated with EndMT in CAD patients, allogeneic kidney transplanted rats and endothelial cells lines. In vivo, Src inhibition remarkably alleviate EndMT and renal allograft interstitial fibrosis in allogeneic kidney transplanted rats. It also had a similar antifibrotic effect in two endothelial cell lines. Mechanistically, the knockout of Src resulted in an augmented AMBRA1‐mediated mitophagy in endothelial cells. We demonstrate that Src knockdown upregulates AMBRA1 level and activates mitophagy by stabilizing Parkin's ubiquitination levels and mitochondrial translocation. Subsequent experiments demonstrated that the knockdown of the Parkin gene inhibited mitophagy in endothelial cells, leading to increased production of Interleukin‐6, thereby inducing EndMT. Consequently, our study underscores Src as a critical mediator of renal vascular EndMT and allograft interstitial fibrosis, exerting its impact through the regulation of AMBRA1/Parkin‐mediated mitophagy.

## Graphical abstract

Our study demonstrated that the activation of Src significantly contributes to the progression of chronic allograft dysfunction, promoting endothelial‐to‐mesenchymal transition (EndMT) and subsequent interstitial fibrosis. We have identified a novel pathway through which Src inhibition mitigates EndMT and fibrosis, underscoring the vital role of AMBRA1‐mediated mitophagy.

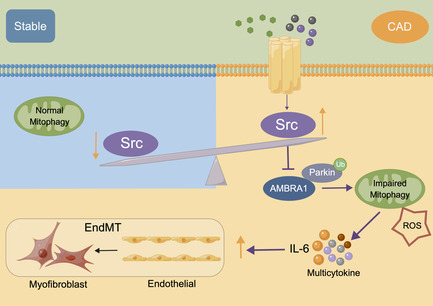

## 1 INTRODUCTION

Kidney transplantation is the preferred treatment for patients with end‐stage renal disease, offering enhanced life quality and a longer life expectancy compared to long‐term dialysis.^1^ However, chronic allograft dysfunction (CAD), marked by an irreversible decline in renal allograft function, remains a major challenge to the long‐term survival of transplanted kidneys.^2^ Acute kidney injury and subsequent CAD, which often lead to transplanted renal fibrosis and graft function deterioration, are influenced by various factors.^3^ Allograft fibrosis, a key aspect of CAD, involves the activation and interaction of multiple profibrotic signalling pathways.^4^ Therefore, the optimal predictor of CAD prognosis is measured by the degree of renal interstitial fibrosis and tubular atrophy termed as interstitial fibrosis and tubular atrophy (IF/TA),^5^ recognized as the ultimate common pathway of progressive renal disease.^4^ During this progression, myofibroblasts synthesize increasing amounts of extracellular matrix (ECM), leading to abnormal deposition of fibrous connective tissues in the allograft.^6^

In kidney transplantation, myofibroblasts maybe originate from various cell types, including mesenchymal cells, bone marrow‐derived cells, fibroblasts, fibrocytes, epithelial cells, endothelial cells and pericytes.^7^ Renal vascular endothelial cells, when injured, could undergo endothelial‐to‐mesenchymal transition (EndMT), contributing to vascular rarefaction, organ fibrosis and dysfunction.^8^ In the process of EndMT, endothelial cells gradually lose their characteristics including decreased CD31 and VE‐cadherin expression and acquire typical myofibroblastic markers such as α‐smooth muscle actin (α‐SMA) and fibronectin (FN).^9^ This transition also alters junction organization, disrupts cell polarity and enhances cell proliferation and migration.^9^ EndMT is crucial in the pathogenesis of various chronic renal diseases and is extensively associated with the initiation and development of organ fibrosis, including heart,^10^ liver^11^ and kidney.^12^ Regulation of EndMT in CAD has also been discussed.^13^ Our previous research has shown that renal vascular EndMT contributes to renal interstitial fibrosis post‐transplantation via the TGF‐β/Smad and Akt/mTOR/p70S6K signalling pathways.^14^

Autophagy, a cellular process for degrading cytoplasmic components, is essential for maintaining kidney homeostasis. Its role in renal fibrosis is complex and not fully understood.^15^ The impact of autophagy on renal fibrosis has been largely investigated while its influence remains elusive. Atg5‐mediated autophagy deficiency in proximal epithelial cells contributed to cell cycle G2/M arrest and renal fibrosis in murine unilateral ureteric obstruction (UUO) model.^16^ Nam also reported autophagy protected distal tubular epithelial cells from developing renal tubulointerstial fibrosis after UUO.^17^ However, activation of autophagy persisted in kidney proximal tubules; consequently, autophagy was considered to have a profibrotic effect in renal interstitial fibrosis during UUO.^18^ Meanwhile, in a rat liver transplantation model, inhibition of autophagy could ameliorate liver allograft survival.^19^ Conversely, autophagy deficiency in endothelial cells could promote Interleukin‐6 (IL‐6)‐induced EndMT and organ fibrosis.^20^, ^21^ Recent studies have begun to explore the role of mitophagy, a specific form of autophagy targeting mitochondria, in organ fibrosis. Di Paola et al. demonstrated that mitophagy, as indicated by increased expressions of PINK1 and Parkin, plays a protective role in liver fibrosis by modulating oxidative imbalance and inflammation. This study also highlighted the anti‐inflammatory effects of mitophagy, including the reduction of pro‐inflammatory cytokines such as IL‐6. These findings suggest a complex interplay between mitophagy, inflammatory responses and fibrosis in organ transplantation, warranting further investigation in the context of kidney transplantation.^22^

Src activation has been revealed critically associated with the development of organ fibrosis.^23^, ^24^ Yan et al. demonstrated that Src was upregulated in both renal interstitial fibroblasts and renal tubular cells of the fibrotic kidney and that Src inhibition with PP1 blocked renal fibroblast activation and attenuated ECM deposition.^25^ Seo et al. also observed that inhibition of SRC with PP2 or saracatinib increased autophagy flux, effectively reduced α‐SMA and Type I collagen expression and attenuated the severity of thioacetamide‐induced liver fibrosis in mice.^24^ Although Src has been reported to be associated with kidney diseases such as acute kidney injury^26^ and diabetic nephropathy,^27^ the role of Src in renal allograft fibrosis is less explored. In this study, we identified the influence of mitophagy on EndMT and the role and molecular mechanism of Src on graft fibrosis after kidney transplantation.

## 2 MATERIALS AND METHODS

### 2.1 Single‐cell transcriptomic analysis

(1) Data import and pre‐processing: The profiles of GSE195718 were downloaded from the GEO database. Raw single‐cell RNA sequencing data were imported into Partek Flow version 10.0.23.0425 (https://www.partek.com/partek-flow/). The data set underwent an initial filtering process to remove potential doublets, as well as low‐quality and dead cells. This was achieved by applying specific criteria: only cells with gene expression counts ranging from 1500 to 15,000, a detected gene count between 400 and 4000, and mitochondrial gene counts less than 20% were retained for downstream analyses. (2) Normalization: The expression matrix was normalized using the binary logarithm of counts per million (CPM) plus one, log2 (CPM + 1), to adjust for variations in sequencing depth and to stabilize the variance across cells. (3) Dimensionality reduction—principal components analysis (PCA): Dimensionality reduction was performed in two steps. The first step involved PCA under default conditions. To determine the number of principal components to retain, a scree plot was utilized, which plotted eigenvalues against the ascending order of principal components. Based on the elbow point observed in the scree plot, the first eight principal components were selected for further analysis. (4) Dimensionality Reduction—t‐SNE: The second step involved creating a two‐dimensional t‐SNE plot for an adequately resolved projection of the data set. This was conducted using the first eight principal components identified from PCA, with a perplexity setting of 40, over 10,000 iterations, and all other parameters set to default. (5) Cluster annotation and visualization: The resultant 2D t‐SNE plot was used to annotate and visualize distinct cellular neighbourhoods. This involved combining graph‐based clustering (utilizing 12 principal components and 1000 iterations per random start, with all other parameters at default) and feature gene expression analysis. The annotated clusters were then visualized on the t‐SNE plot, providing insights into the cellular heterogeneity and underlying biological processes in the sample. The identification of hub genes was performed using Cytoscape software v3.10.1. GO and KEGG pathway enrichment analysis were conducted using DAVID (https://david.ncifcrf.gov/tools.jsp).

### 2.2 Sample collection

Tissues obtained from recipients who underwent allograft biopsy or resection were allocated into the CAD group and the stable renal graft function (Stable) group based on their clinical manifestation and the 2017 Banff Classification of Renal Allograft Pathology.^28^ Among them, 30 recipients were assigned into the Stable group while 30 recipients were diagnosed with CAD. The demographic characteristics of the two groups are summarized in Table 1.

**TABLE 1:** Baseline characteristics of the CAD and Stable groups.

Clinical variables	CAD group	Stable group	*p*‐value
Case number (*n*)	30	30	NS
Age (years, mean ± SD)	35.59 ± 3.09	38.61 ± 2.41	NS
Gender (male/female)	18/12	20/10	NS
BMI (kg/m^2^, mean ± SD)	22.64 ± 4.71	23.21 ± 4.2	NS
Transplant duration (years, range)	9.1 (6.5–13)	3.4 (2.3–4.8)	<0.001
Primary/secondary transplant	30/0	30/0	NS
PRA before renal transplant (%)	0	0	NS
Donor source			NS
Living related	25	21	
Cadaveric	5	9	
Immunosuppressive regimen			NS
Prednisone + MMF + Tac	20	22	
Prednisone + MMF + CsA	10	8	
Biochemical parameters			
Serum creatinine (μmol/L, mean ± SD)	418.1 ± 20.8	93.37 ± 10.68	<0.001
eGFR^a^ (min/1.73 m^2^, mean ± SD)	24.71 ± 2.88	76.79 ± 6.23	<0.001

Abbreviations: BMI, body mass index; CAD, chronic allograft dysfunction; CsA, cyclosporine A; eGFR, estimated glomerular filtration rate; MMF, mycophenolate mofetil; NS, no significance; PRA, panel reaction antibody; Tac, tacrolimus.

^a^ eGFR was estimated by the Cockcroft–Gault formula: eGFR = (140 − age) × weight/72 × serum creatinine × (0.85 if female).

### 2.3 Establishment of rat kidney transplantation model

Lewis (LEW/Crl; LEW) rats and F344 (F344/DuCrl; CDF) rats (6–8 weeks of age) were purchased from Charles River Laboratories (Beijing, China). The animals followed the standards of Nanjing Medical University and the ethical guidelines issued by the US National Institutes of Health. All animal experiments procedures complied with the guidelines of the Institutional Animal Care and Use Committee at Nanjing Medical University.

Orthotopic left kidney transplantations were carried out between Lewis and F344 rats.^29^ Rats were divided into four groups after renal transplantation, Donor is Lewis and recipient is Lewis in Syn group, Donor is F344 and recipient is Lewis in Allo group, Syn + PP1 group is Lewis to Lewis with the administration of PP1. Allo + PP1 group is F344 to Lewis with the administration of PP1. Orthotopic renal removal were performed 10 days after the surgery. Different concentrations of PP1 (2 mg/kg) were given to the rats in the Allo + PP1 group. PP1 was dissolved in sterile phosphate buffer saline and administered to the rats by gavage once a day. Cyclosporine A (5 mg/kg, qd, ip; Neoral, Novartis, Switzerland) was used to avoid acute rejection for 14 days.

### 2.4 Tissue harvest

In our previous investigations,^29^ we conducted histopathological and renal function assessments of transplanted rat kidneys at 8, 12 and 16 weeks post‐transplantation. These studies revealed that the degree of fibrosis was most pronounced at 16 weeks post‐transplantation, and the differences in fibrosis following interventions were most significant at this time point. Consequently, the kidneys were harvested from transplant rats at 16 weeks. Allograft tissues were embedded with paraffin and fixed in 10% neutral formalin for histological and Immunohistochemistry (IHC)/IF staining. The remaining tissues were stored at −80°C for analysis of protein and RNA.

### 2.5 Cell culture, treatment and siRNA/shRNA transfection

HUVECs and HRGECs were obtained from ScienCell Research Laboratories (4000, 8000, ScienCell; Santiago, USA) and cultured with F12k medium (1001, ScienCell; Santiago, USA), containing 10% fetal bovine serum (0025, ScienCell; Santiago, USA), 5 mL endothelial cell growth supplement (1052, ScienCell; Santiago, USA) and 5 mL penicillin (10,000 U/mL)/streptomycin (10,000 μg/mL) solution (0503, ScienCell; Santiago, USA). IL‐6 was incubated with cells for different times (0, 6, 12 and 24 h) or with different concentration (0, 1, 10 and 20 ng/mL) for 24 h. The concentration of 3‐MA for autophagy inhibition in 10 mM in vitro experiments.

siRNA and shRNA were constructed by KaiJi Biotechnology Co. Ltd. (Jiangsu, China). Then, 50 nM of Parkin siRNA and control siRNA were transfected using Lipofectamine 3000 kit (Invitrogen, USA) based on the manufacturer's protocol. After transfection for 24 h, HUVECs were harvested for total protein extraction and IF staining. For shRNA transfection, HUVECs were transfected with the designated AMBRA1 shRNA plasmids by using shRNA plasmid transfection reagent (Santa Cruz).

### 2.6 Enzyme‐linked immunosorbent assay

Quantification of serum IL‐6 levels and cytokine IL‐1I levels were detected by ELISA kit from MUTISCIENCES (70‐EK306/3‐96, 70‐EK111‐96, Beijing, China). Cytokines (IL‐1β, IL‐2, IL‐4, IL‐5, IL‐6, IL‐10, IL‐17A, TNF‐α, TGF‐β1) were measured by PeliKine Compact human ELISA kit (BioLegend; San Diego, USA), based on the manufacturer's instructions.

### 2.7 Histopathology

H&E and Masson's trichrome staining assays were conducted as described previously.^29^ The extent of fibrotic area was used to evaluate the severity of CAD, then quantitatively analysed by Image‐Pro Plus 3.0 (Media Cybernetics, Rockville, MD).

### 2.8 IHC staining assay

The expression and distribution of FN and α‐SMA were measured by IHC staining assay. The preparation of kidney tissues and staining methods for IHC assay was consistent with this previous study.^14^ Primary antibodies used in this study were as follows: anti‐α‐SMA (1:100; Abcam, UK), anti‐FN (1:500; BD, USA).

### 2.9 IF staining assay

Renal allograft tissues and cells were fixed with 4% formaldehyde solution, penetrated with 0.1% Triton X‐100 for 1 h, blocked with 5% goat serum for 1 h, and then incubated with primary antibodies at 4°C overnight. Subsequently, secondary antibodies and Dapi were used as described by our previous study.^14^ Primary antibodies used in this study were as follows: anti‐FSP‐1 (1:100; CST, USA); anti‐p‐Src (1:100; CST); anti‐MAP1LC3 (1:100; CST); anti‐COX‐IV (1:100; CST); anti‐CD31(1:100; Abcam); anti‐α‐SMA (1:100; Abcam); anti‐AMBRA1 (1:100; Abcam); anti‐Parkin (1:100; Abcam); anti‐Ub (1:100; Abcam).

### 2.10 Western blot and coimmunoprecipitation assay

The procedures of western blot and coimmunoprecipitation analysis were shown in our previous study.^29^ Primary antibodies were as follows: anti‐Src (1:1000; CST); anti‐p‐Src^Tyr416^ (1:1000; CST); anti‐AMBRA1 (1:1000; CST); anti‐Parkin (1:1000; CST); anti‐Pink1 (1:1000; CST); anti‐ubiquitin (1:1000; CST); anti‐GAPDH (1:10000; BD); anti‐β‐ACTIN (1:10000; BD); anti‐COX‐IV (1:1000; CST); anti‐LC3I‐II (1:1000; CST); anti‐TOMM20 (1:1000; CST); anti‐SQSTM1/p62 (1:1000; CST); anti‐CD31(1:1000; Abcam); anti‐a‐SMA (1:1000; Abcam); anti‐VE‐cadherin (1:1000; Abcam); anti‐IL‐6 (1:5000; Abcam); anti‐FN (1:10,000; BD). All secondary antibodies for western blot analysis from Thermo Fisher Scientific. Antigen–antibody complexes on the membranes were detected with an enhanced chemiluminescence kit from Thermo Scientific. For quantification, protein bands were analysed with ImageJ software.

### 2.11 Quantitative real‐time polymerase

Total RNAs were extracted from tissues and cells with the RNA Extraction Kits (TIANGEN, Beijing, China). Detailed steps are described in our previous paper.^29^ Gene expressions were measured by real‐time PCR assay (Vazyme, Nanjing, China) and a DNA Engine Opticon 2 System (BioRad Laboratories, Hercules, CA, USA). Every experiment was repeated at least three times. The specific primers used were as follows:

RNF2: 5′‐CAAACGGAACTCAACCATTAAGC‐3′ (F)

5′‐CCACTTCTAAGGGCTGTGATG‐3′ (R)

MARCH5: 5′‐GTCCAGTGGTTTACGTCTTGG‐3′ (F)

5′‐CCGACCATTATTCCTGCTGC‐3′ (R)

### 2.12 Flow cytometry

MitoSOX and JC‐1 staining was performed according to the manufacturer's instructions (Beyotime, China). JC‐1 dye was employed to evaluate mitochondrial membrane potential. In HUVECs with high mitochondrial membrane potential, JC‐1 aggregates and emits red fluorescence, whereas in apoptotic or damaged cells with low membrane potential, JC‐1 remains in its monomeric form, emitting green fluorescence. The cells were then washed and resuspended for flow cytometric analysis. MitoSOX Red for ROS: To assess mitochondrial ROS production, cells were stained with MitoSOX Red, a mitochondrial superoxide indicator. The cells were incubated with MitoSOX Red reagent for 30 minutes at 37°C, protected from light. Following incubation, cells were washed and resuspended in warm buffer for immediate analysis by flow cytometry.

### 2.13 Bulk RNA sequencing

Total RNA was extracted from HUVECs using the TRIzol reagent (Invitrogen), following the manufacturer's protocol. RNA purity and concentration were assessed using the NanoDrop ND‐1000 spectrophotometer (Thermo Fisher Scientific), and RNA integrity was verified by agarose gel electrophoresis. For bulk RNA sequencing, 1 μg of total RNA from each sample was used for library preparation. The RNA samples were first treated with DNase I to remove any potential DNA contamination. Following this, the RNA was fragmented and reverse transcribed into cDNA using random hexamer priming. The cDNA was then subjected to end‐repair, A‐tailing and adapter ligation. The libraries were amplified using PCR and validated for quality and quantity using the Agilent 2100 Bioanalyzer (Agilent Technologies) and Qubit 2.0 Fluorometer (Life Technologies). High‐throughput sequencing was performed on an Illumina platform. Raw data were processed for quality control and the reads were aligned to the human reference genome. Differential gene expression analysis was conducted using appropriate bioinformatics tools.

### 2.14 Statistical analysis

All data were presented as mean ± standard deviation calculated from at least three independent experiments. Comparison between groups were performed with one‐way analysis of variance. Multiple comparisons were made using Tukey's test. Pearson correlation coefficient was used for the correlation analysis. All statistical analyses were conducted using the SPSS statistical software version 25, with a *p*‐value <0.05 considered statistically significant.

## 3 RESULTS

### 3.1 Single‐cell sequencing analysis revealed Src kinase's central role in CAD and interstitial fibrosis of transplanted kidneys

**FIGURE 1:**
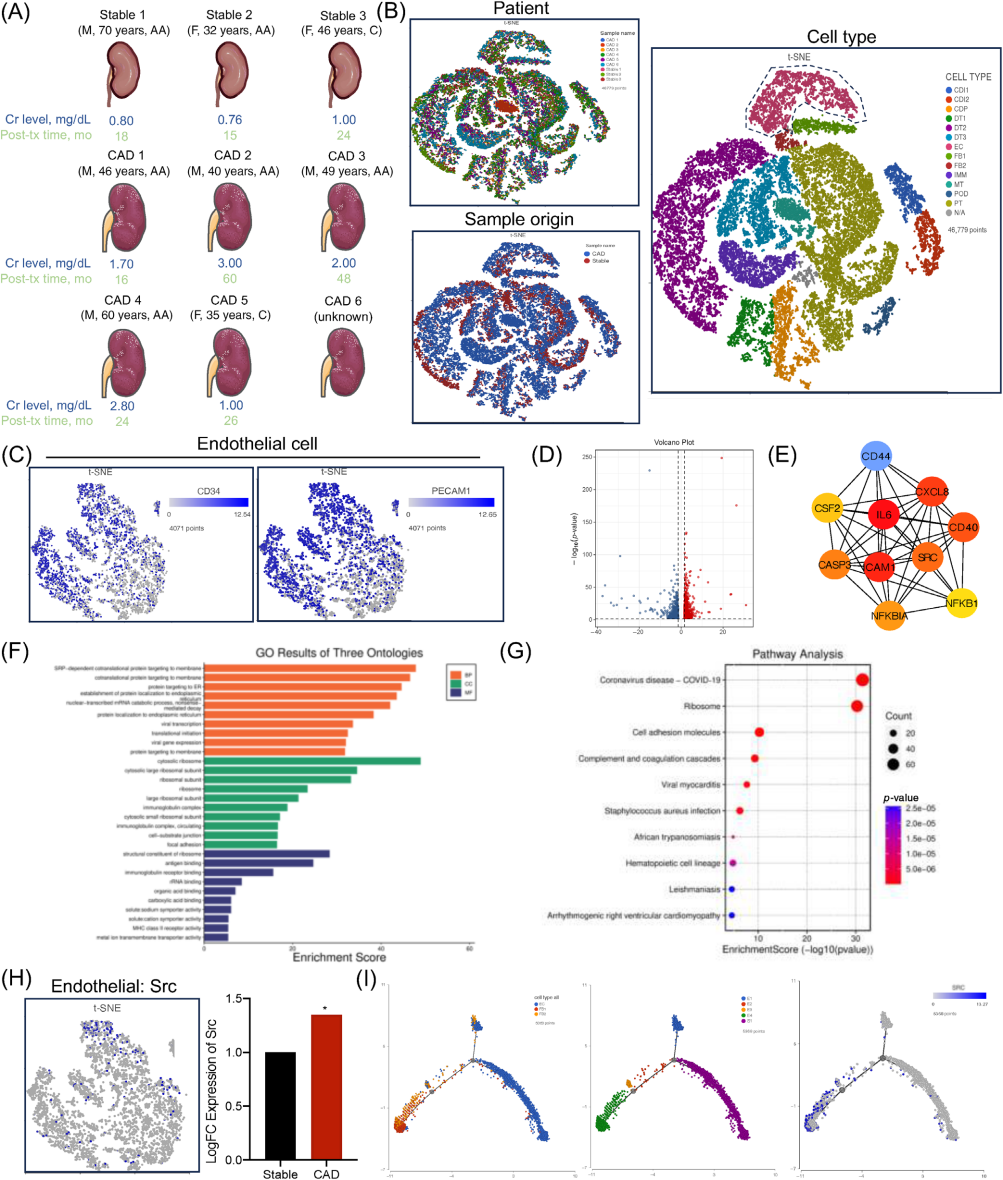
Single‐cell sequencing analysis revealed Src kinase's central role in CAD and interstitial fibrosis of transplanted kidneys. (A) Overview of clinical information and single‐cell RNA sequencing data from six CAD patients and three stable renal transplant recipients from GSE195718. (B) t‐SNE visualization of 41,893 cells post‐quality control, depicting the distribution of cell types from nine clinical patients. CAD and stable groups are colour‐coded for differentiation. (C) t‐SNE showing the annotation of endothelial cells using markers CD34 and PECAM1. (D) Volcano plot illustrating 2632 differentially expressed genes in endothelial cells, with upregulated genes in red and downregulated genes in blue. (E) Network visualization of hub genes and its protein–protein interaction analysis among differentially expressed genes, highlighting central genes such as Src, IL‐6 and NF‐κB1. (F, G) GO analysis (F) and KEGG analysis (G) of DEGs in endothelial cells between the CAD and stable group. (H) t‐SNE showing the scaled expression of Src in endothelial cells. (I) Pseudo‐time trajectory analysis of endothelial cells transitioning to fibroblasts and the dynamic changes of Src expression between the CAD and stable group. AA, African American; C, Caucasian; CAD, chronic allograft dysfunction; Cr, creatinine; DEGs, differentially expressed genes; F, female; GO, Gene Ontology; IL‐6, Interleukin‐6; KEGG, Kyoto Encyclopaedia of Genes and Genomes; M, male; mo, month; NF‐κB1, nuclear factor kappa B subunit 1.

In our comprehensive analysis, we retrieved single‐cell sequencing data of six CAD patients and three stable renal transplant recipients from the Gene Expression Omnibus (GEO) public database, data set GSE195718. This included associated clinical information, as shown in Figure 1A. The data set focused on single‐cell RNA sequencing of non‐fibrotic and fibrotic human renal allografts, encompassing a total of 41,893 cells after quality control and doublet removal (informative details are shown in the Supplementary Figure 1A and Methods). These cells were visualized using the T‐ Stochastic Neighbour Embedding (t‐SNE) method, which facilitated a better understanding of the complexity and diversity of cell types present in the data set. Different cell populations were colour‐coded for easy identification and analysis. Figure 1B displays the distribution of nine clinical patients, as well as the CAD and stable groups in the t‐SNE plot, primarily distinguishing cell types through unique cellular markers.

The identified cell types included Collecting Duct Intercalated, Collecting Duct Principal, Distal Tubular, Endothelial, Fibroblast, Immune, Mixed Tubular, Podocyte, Proximal Tubular and an unknown cell type, provisionally labelled as UKN1. Our primary focus was on endothelial cells, echoing their significant role in vascular health and pathology, as revealed in our previous studies. In Figure 1C, endothelial cells were annotated using CD34 and PECAM1. Subsequent differential expression analysis of these cells revealed a total of 2632 differentially expressed genes, graphically represented in a volcano plot in Figure 1D, with upregulated genes marked in red and downregulated genes in blue. This visualization provided a clear representation of gene expression differences, aiding in a deeper understanding of the molecular landscape of endothelial cells in our patient cohort. Further analysis of these differentially expressed genes was conducted through the STRING database to calculate the protein–protein interaction network. This step helped us understand how proteins encoded by these genes interact and influence cellular processes. We used Cytoscape to visualize the interaction network and identify hub genes. Genes were colour‐coded based on their expression levels. Notably, several genes, including Src, IL‐6 and nuclear factor kappa B subunit 1 (NF‐κB1), were found to occupy central positions in the differential gene network. These genes are renowned for their crucial functions in immune response and inflammation processes, suggesting their potential significant role in the pathophysiological alterations of endothelial cells in CAD patients and kidney transplanted recipients (Figure 1E).

Additionally, we conducted Gene Ontology (GO) functional and Kyoto Encyclopaedia of Genes and Genomes (KEGG) pathway analyses. The GO functions included co‐translational protein targeting to membrane, cytosolic ribosome, antigen binding and immunoglobulin receptor binding. Pathways were primarily enriched in COVID‐19 and cell adhesion molecules (Figure 1F,G). Furthermore, as shown in Figure 1H, Src was significantly upregulated in endothelial cell subgroups, a finding consistent with our earlier protein–protein interaction results and further supporting the significance of our observations. We also examined Src expression in various cell types; results and *p*‐value are shown as in Supplementary Figure 1B. Considering our previous studies that emphasized the critical involvement of EndMT in the interstitial fibrosis process of transplanted kidneys, we expanded our focus to a more comprehensive single‐cell sequencing analysis, encompassing endothelial cells, fibroblasts and myofibroblasts. Our aim was to use pseudo‐time trajectory analysis to discern whether Src plays a central role in this transition. In Figure 1I, we depicted different cell types through colour coding: blue for endothelial cells, with red and yellow representing different fibroblast groups. For analytical ease, endothelial cells were designated as the starting state (S1). As we traced the trajectory, these cells evolved through four consecutive stages, marked as expanded states E1–E4.

Our findings were striking. Src gene expression levels were elevated in endothelial cell state E4 and the identified fibroblast groups (Fibroblast 1 and Fibroblast 2). These patterns provide convincing evidence suggesting that Src might play a key role in the progression of renal interstitial fibrosis in allogeneic kidney transplantation. This discovery, combined with our previous research, highlights the potential therapeutic value of targeting Src in alleviating transplanted renal fibrosis in recipients.

### 3.2 Upregulated Src associated with EndMT in CAD: Histological evidence from transplanted kidneys analysis

**FIGURE 2:**
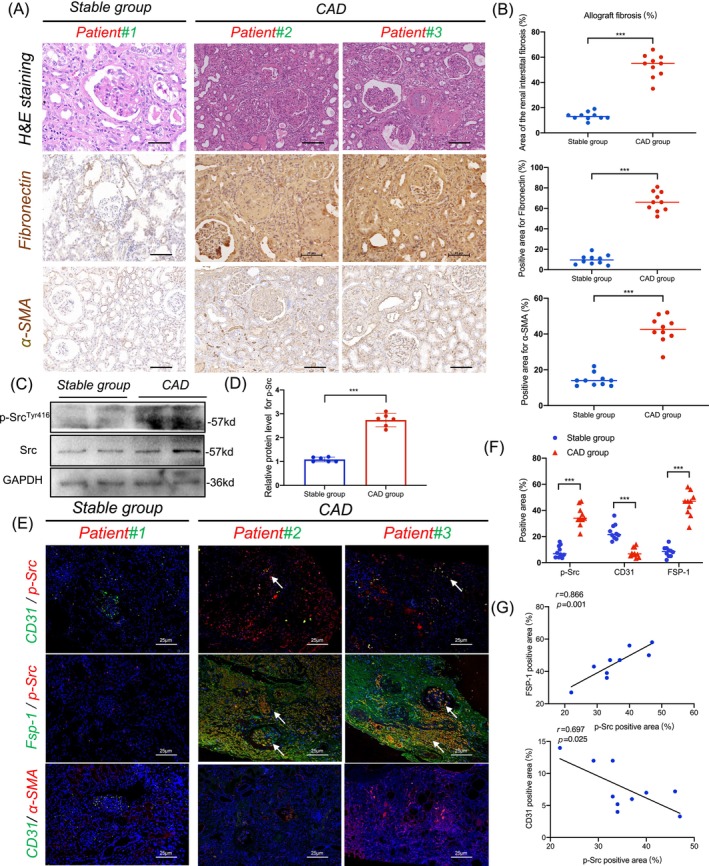
Src is activated in endothelial cells from chronic renal allograft dysfunction (CAD) patients. (A) Representative images of H&E and IHC staining in renal allograft tissues in the stable group and CAD group. (B) Statistical graphs of fibrosis area, fibronectin and α‐SMA expression in kidney allograft tissues from the stable group and CAD group (*n* = 5) (bar = 20 μm). (C, D) Western blot analysis (C) and densitometric quantification (D) of Src and *p*‐Src expression in transplanted kidney tissues from the stable (*n* = 5) and CAD (*n* = 5) groups. (E, F) Representative immunofluorescence staining images (E) and statistical graphs (F) of CD31, p‐Src, FSP‐1 and α‐SMA in transplanted kidney tissues from the stable group and CAD group (*n* = 5) (bar = 20 μm). (G) Pearson correlation analysis between FSP‐1, CD31 expression and p‐Src expression. Data were presented as mean ± SEM. *p < 0.05; **p < 0.01; ***p < 0.001. CAD, chronic allograft dysfunction; IHC, immunohistochemistry; α‐SMA, α‐smooth muscle actin.

To further delve into the pathophysiological changes associated with CAD in the context of kidney transplantation, we undertook a comprehensive histological comparison between patients diagnosed with CAD and a stable control group. Representative pathological change of HE staining is displayed in Figure 2A. Haematoxylin and eosin (H&E) staining revealed normal glomerular and tubular structures in the stable group, and more glomerulosclerosis, arteriosclerosis and IF/TA as chronic lesions in CAD group. Histological staining illuminated notable disparities in collagen fibre deposition between the two cohorts. The CAD patient group (*n* = 10) manifested markedly more extensive collagen fibre accumulation than their control counterparts (*n* = 10). This heightened collagen presence emblematically denotes the pathological characteristics commonly seen in renal allograft fibrosis. Immunohistochemical techniques substantiated our initial findings, revealing augmented expressions of α‐SMA and FN in the CAD group, both being hallmark indicators of fibrotic changes (Figure 2A,B). Given the central role of Src in EndMT, as suggested by our prior single‐cell sequencing findings, we incorporated an additional western blot assay step to detect Src. Phosphorylated Src (p‐Src^Tyr416^) is an activated form of Src. Results showed that the CAD group exhibited a significant surge in p‐Src‐positive cells when juxtaposed against the control group (Figure 2C,D). To further cement our observations, particularly in the realm of EndMT, we embarked on a double immunofluorescence (IF) staining approach. This targeted the detection of EndMT markers within the biopsy specimens from both study groups. We observed a pronounced reduction in the endothelial cell marker, CD31, within the CAD group as compared to the controls. Concurrently, an amplified presence of the myofibroblast marker, fibroblast‐specific protein 1 (FSP‐1), was conspicuous in the CAD samples. Intriguingly, the CAD group also demonstrated a positive correlation between FSP‐1‐positive cells and the expanse of p‐Src‐positive cells. On the contrary, a negative correlation emerged between CD31 and p‐Src in the stable control group (Figure 2E–G). In addition, our findings also indicate that Src does not increase during acute rejection episodes in patients with antibody‐mediated rejection (ABMR) and T‐cell‐mediated rejection. This suggests that Src may play a more significant role in fibrogenesis rather than in the acute rejection process itself (Supplementary Figure 2A). Collectively, these findings underscore a potentially intricate role of Src activation in mediating EndMT. This, in turn, might have profound implications in exacerbating transplanted renal fibrosis, emphasizing the therapeutic potential of targeting Src in ameliorating these fibrotic changes.

### 3.3 Blocking Src kinase attenuated renal allograft fibrosis and improved allograft function

**FIGURE 3:**
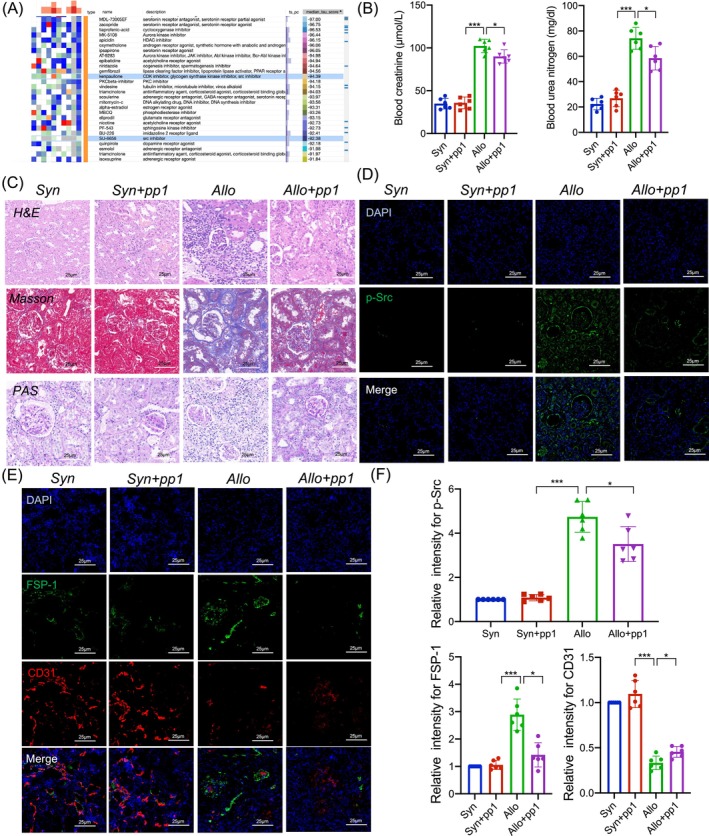
Ablation of Src alleviates EndMT and allograft fibrosis. (A) Data mining of gene expression profiles and drug screening in the Connectivity Map database. (B) Renal function indicators of kidney transplanted mice with or without PP1 treatment. (C) Representative images of H&E, Masson and PAS staining in renal allograft tissues from the Syn, Syn with PP1, Allo and Allo with PP1 groups (*n* = 6). (D) Representative immunofluorescence staining images of p‐Src expression in transplanted kidney tissues from the four groups (*n* = 6). (E) Representative immunofluorescence staining images of colocalization of CD31 and FSP‐1 in transplanted kidney tissues from the four groups (*n* = 6) (bar = 20 μm). (F) Statistical graphs of p‐Src, CD31 and FSP‐1 in transplanted kidney tissues from the four groups (*n* = 6). Data were presented as mean ± SEM. *p < 0.05; **p < 0.01; ***p < 0.001. EndMT, endothelial‐to‐mesenchymal transition.

Building upon our prior investigations, we explored the therapeutic potential of differential gene networks in endothelial cells from CAD patients and renal transplant recipients. We specifically examined the associations between differentially expressed genes (DEGs) and prospective drug therapies. To facilitate this, DEGs were input into the Connectivity Map (CMAP) database, which correlates gene expression patterns with small molecule effects, assigning a unique score to depict the relationship strength. Scores nearing 1 indicate a positive correlation, suggesting compatibility between the drug and the gene of interest. Our analysis highlighted two small molecule inhibitors, Kenpaullone and SU‐6656, which are known to inhibit Src family kinases (Figure 3A). Their involvement points to a possible therapeutic application in treating endothelial dysfunction in renal allograft fibrosis.

PP1, a selective inhibitor of the Src family of kinases, was administered to rats to suppress Src expression and examine its effects on renal graft function. To ensure the optimal and safe long‐term application of PP1, we conducted a series of cytotoxicity and efficacy tests. As a result, the ideal dosage was determined to be 2 mg/kg in 50 μL dimethyl sulfoxide. Recipient rats given this dose of the drug improved renal function and pathological progression. In the Allo group, there was a notable increase in blood urea nitrogen (BUN) and creatinine levels, indicating of renal impairment, compared to the Syn group (Figure 3B, *p* < 0.05). Treatment with PP1 significantly reduced BUN and creatinine levels in the Allo + PP1 group, suggesting improved renal allograft function (Figure 3B). Over the course of 16 weeks post‐transplantation of kidney, renal allograft fibrosis increased, with the affected area constituting approximately 70% of the allograft, as demonstrated in Figure 3C. Moreover, IF assay showed an increase in phosphorylated Src (p‐src) level in the Allo group, which was significantly attenuated by PP1 treatment, as evidenced in Figure 3D. Similarly, IF results showed that the administration of PP1 alleviated EndMT progression (Figure 3E,F). Further analysis using IF double staining assay showed that the Allo + PP1 group had a marked reduction in FSP‐1 expression and an increase in CD31 expression, implying an attenuation of renal vascular EndMT (Figure 3E). In addition, PP1 significantly decreased the co‐expression of FSP‐1 and CD31 in endothelial cells within the renal vascular and glomerular structures, enhancing endothelial cell function and further supporting the therapeutic efficacy of PP1.

### 3.4 Src inhibition attenuated TGF‐β1‐mediated EndMT and cell migration in human umbilical vein endothelial cells and human renal glomerular endothelial cells

Our previous research established the significant role of TGF‐β1‐induced EndMT in the progression of renal fibrosis in allogeneic kidney transplantation. To investigate the effects of TGF‐β1 on Src, we stimulated human umbilical vein endothelial cells (HUVECs) with varying concentrations and durations of TGF‐β1 and monitored changes in p‐Src and markers of EndMT. We observed that TGF‐β1 at a concentration of 20 ng/mL significantly increased the expression of FN and α‐SMA, while the endothelial cell marker CD31 was most substantially decreased. Concomitantly, p‐Src level also escalated in line with the increase in EndMT. Hence, we continued to stimulate endothelial cells with this TGF‐β1 concentration for different durations. Immunoblotting assay results showed that the markers of EndMT peaked at 24 h without further notable increases, while p‐Src was activated as early as 3 h, reaching its peak at 24 h (Supplementary Figure 3A–C).

**FIGURE 4:**
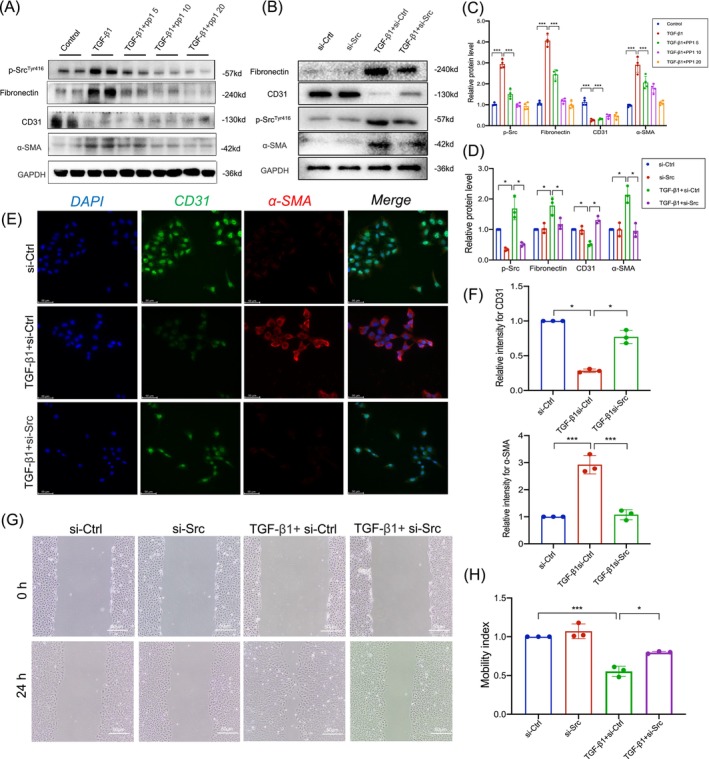
Src inhibition attenuated TGF‐β1‐mediated EndMT, cell migration and invasion in endothelial cells. (A, B) HUVECs were treated with 20 ng/mL TGF‐β1 and different concentration PP1 for 24 h. Western blot analysis (A) and densitometric quantification (C) of p‐Src, fibronectin, CD31 and α‐SMA expression (*n* = 3). (C, D) HUVECs were transfected with si‐Ctrl or si‐Src for 24 h. After transfection, HUVECs were treated with 20 ng/mL TGF‐β1 for 24 h. Western blot analysis (B) and densitometric quantification (D) of p‐Src, fibronectin, CD31 and α‐SMA expression (*n* = 3). (E, F) Representative images (E) and statistical graphs (F) for immunofluorescence staining of CD31 and α‐SMA in HUVECs (*n* = 3) (bar = 50 μm). (G, H) Quantitative analyses results of the motility index of HUVECs treated with TGF‐β1 and/or Src knockdown, the motility index has determined by the formula ‘migration cell number of control group/migration cell number of the other treatment group’ (*n* = 3) (bar = 50 μm). Data were presented as mean ± SEM. *p < 0.05; **p < 0.01; ***p < 0.001. EndMT, endothelial‐to‐mesenchymal transition; HUVECs, human umbilical vein endothelial cells; α‐SMA, α‐smooth muscle actin.

Subsequently, we treated HUVECs with 20 ng/mL TGF‐β1 and various concentrations of PP1 to simulate in vivo conditions. PP1 significantly reduced TGF‐β1‐induced protein expression of α‐SMA and FN, while partially restoring CD31 expression (Figure 4A–D). Similar effects were achieved by the knockdown of Src with small interfering RNA. Furthermore, indirect IF staining of α‐SMA and CD31 showed that PP1 could inhibit the EndMT progression induced by TGF‐β1 in endothelial cells (Figure 4E,F). The similar results were obtained using human renal glomerular endothelial cells (HRGECs) primary cell line (Supplementary Figure 3D–I).

Additionally, we evaluated changes in cell migratory capabilities, which are highly associated with EndMT, through functional assays. The wound healing assay was employed to assess migratory capacity following treatment with TGF‐β1 and PP1 or in their absence. In these assays, TGF‐β1 notably increased the chemokinetic motility of endothelial cells, an effect that was reversed by PP1 (Figure 4G,H). These findings support the hypothesis that treating endothelial cells with PP1 can ameliorate TGF‐β1‐induced EndMT.

### 3.5 Mitophagy was activated following Src inhibition in endothelial cells

**FIGURE 5:**
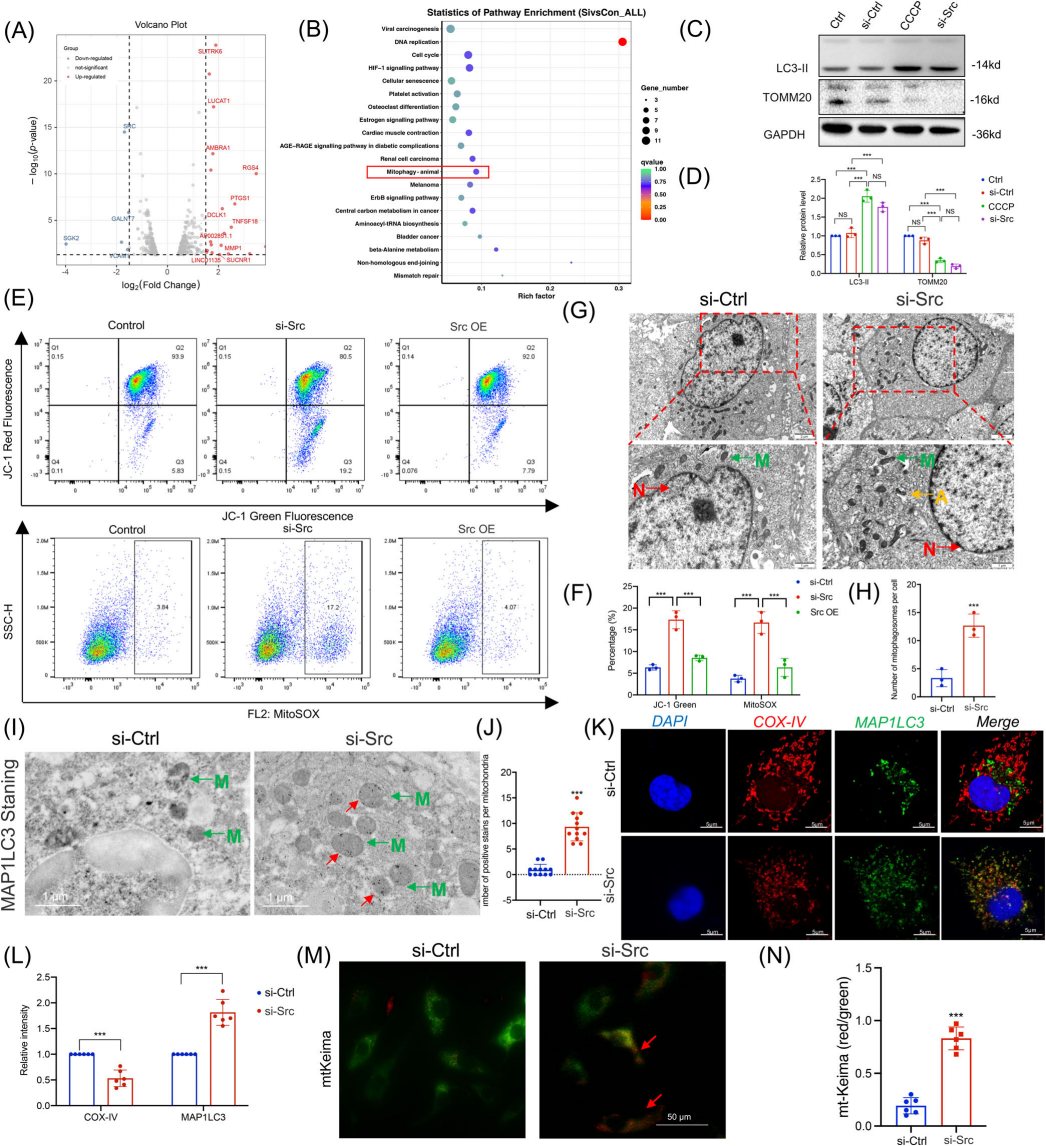
Mitophagy was activated following Src inhibition in endothelial cells. (A) The RNA sequencing results of differential genes in HUVECs transfected with si‐Ctrl or si‐Src for 24 h (*n* = 3). (B) KEGG pathway analyses results of the top 20 KEGG‐enriched gene pathways base on RNA sequencing results. (C, D) Western blot analysis (C) and densitometric quantification (D) of p62, LC3‐II and TOMM20 expression between the si‐Ctrl and si‐Src groups (*n* = 3). (E, F) Representative images of MitoSox and JC‐1 intensities in the differentially treated HUVECs (E) and flow cytometric quantification of distribution ratio (F) (*n* = 3). (G, H) Electron microscopy was applied to evaluate the occurrence of mitophagy (*n* = 3) (bar = 1 μm). Yellow arrow: mitophagy of autolysosome phase; green arrow: mitochondria; red arrow: nucleus. (I, J) Immunoelectron microscopy of MAP1LC3 (*n* = 12) (bar = 1 μm). Red arrow: stain the positive expression area; green arrow: mitochondria. (K, L) Representative images (K) and statistical graphs (L) for immunofluorescence staining of COX‐IV and MAP1LC3 in HUVECs (*n* = 6) (bar = 5 μm). (M, N) Representative confocal images (M) and statistical graphs (N) of mtKeima in HUVEC with or without treatment with si‐Src (*n* = 6) (bar = 50 μm). HUVECs, human umbilical vein endothelial cells; KEGG, Kyoto Encyclopaedia of Genes and Genomes.

To further elucidate the role of Src in regulating EndMT, we conducted next‐generation RNA sequencing on HUVECs treated with or without PP1. The comparison between the PP1‐treated group and the control group revealed 2321 differentially expressed genes, including 891 genes with reduced expression such as Src, depicted in blue and 1430 genes with increased expression including AMBRA1, MMP1 and RGS4, indicated in red (Figure 5A). A total of 903 differentially expressed genes had a log fold change (logFC) less than −2 and a *p*‐value less than 0.05.

KEGG pathway analysis of the RNA‐seq data identified 127 pathways, with the top 20 most significantly enriched pathways listed, including those related to DNA repair, cell cycle, HIF‐1 hypoxia signalling and mitophagy, sorted by *p*‐value (Figure 5B). Our previous research demonstrated that autophagy played a crucial role in the regulation of EndMT, leading us to explore whether Src could exert its effects through the activation of mitophagy. The KEGG enrichment results of mitophagy are shown in Supplementary Figure 4. To validate the RNA‐seq data, we performed western blot analysis to determine if the knockdown of Src could activate mitophagy. We assessed the protein levels of LC3‐II, and Tomm20, which are markers of mitophagy. LC3‐II is associated with autophagosome membranes, and its levels increase during autophagy. Tomm20, a mitochondrial outer membrane protein, is degraded by mitophagy. The knockdown of Src significantly activated mitophagy, as indicated by the changes in these markers (Figure 5C,D).

The occurrence of mitophagy brings about changes in mitochondrial function, so we also examined mitochondrial function and oxidative stress markers. We used flow cytometry with JC‐1 and MitoSox probes to detect mitochondrial membrane potential and mitochondrial reactive oxygen species (ROS), respectively. The signal of JC‐1 aggregates is a marker of mitochondrial polarization (JC‐1 red), while cells containing only JC‐1 monomers indicate mitochondrial depolarization (JC‐1 green). Flow cytometry results suggested an increase in JC‐1 green fluorescence, indicating mitochondrial depolarization after Src knockdown, while overexpression of Src via plasmid did not result in significant changes compared to the control group. MitoSOX is used to assess mitochondrial ROS, and we found an increase in ROS levels after Src knockdown (Figure 5E,F). These results represent mitochondrial damage and dysfunction following Src knockdown.

Electron microscopy is the gold standard for observing mitophagy, and our study analysed the significant features of mitophagosomes and autophagosomes through transmission electron microscopy. We observed that autophagosomes engulfed autophagic vesicles and mitochondria following si‐Src treatment, which also showed mitochondrial cristae damage via electron microscopy analysis (Figure 5G,H). Binding of MAP1LC3 to mitochondria was further confirmed by immunogold electron microscopy staining (Figure 5I,J).

To further confirm the impact of Src knockdown on mitophagy, we performed co‐localization staining of endothelial cells for the mitochondrial protein Cox‐IV and the autophagosome marker MAP1LC3. IF co‐localization staining results consistently confirmed that si‐Src treatment increased the co‐localization of mitochondrial protein Cox‐IV with the autophagy marker MAP1LC3, also indicating the activation of mitophagy (Figure 5K,L). Moreover, mtKeima assay as well indicated that si‐Src increased mitophagy index (Figure 5M,N).

### 3.6 Regulation and impact of AMBRA1 in mitophagy inhibited by Src activation

**FIGURE 6:**
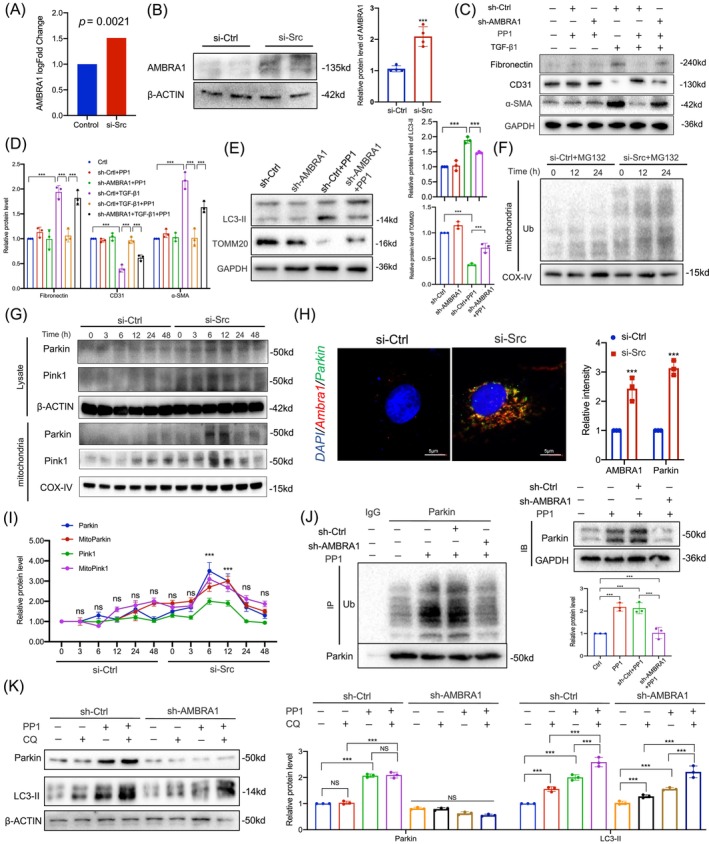
Regulation and impact of AMBRA1 in Mitophagy Inhibited by Src activation. (A) Analysis of AMBRA1 gene expression by RNA sequencing. (B) Western blot analysis and densitometric quantification of AMBRA1 expression validation (*n* = 4). (C) HUVECs were transfected with shRNA control or sh‐AMBRA1 for 24 h, then were starved overnight and treated with/without TGF‐β1 (20 ng/mL), PP1 (20 ng/mL) for 24 h. Western blot analysis (C) and densitometric quantification (D) of fibronectin, CD31 and α‐SMA expression in different groups (*n* = 3). (E) Western blot analysis and densitometric quantification of LC3‐II and TOMM20 expression in different groups (*n* = 3). (F) Western blot analysis of mitochondrial ubiquitination between the control and si‐Src group treated with 10 μM MG132 for 6 h (*n* = 3). (G, I) Western blot analysis (G) and densitometric quantification (I) of Parkin and Pink1 in total cell lysates and mitochondria of HUVECs between the control and si‐Src group (*n* = 3). (H) Representative images (E) and statistical graphs (F) for immunofluorescence staining of AMBRA1 and Parkin in HUVECs (bar = 5 μm) (*n* = 3). (J) Co‐immunoprecipitation assay for the ubiquitination of Parkin in different groups (*n* = 3). (K) HUVECs were transfected with shRNA control or sh‐AMBRA1 for 24 h, then were starved overnight and treated with/without CQ (40 ng/mL), PP1 (20 ng/mL) for 24 h. Western blot analysis and densitometric quantification (K) of Parkin and LC3‐II expression in different groups (*n* = 3). Data were presented as mean ± SEM. *p < 0.05; ***p < 0.001. HUVECs, human umbilical vein endothelial cells; NS, non‐significant; α‐SMA, α‐smooth muscle actin.

Based on KEGG enrichment analysis, AMBRA1 was found to be upregulated in the mitophagy pathway. Figure 6A details the fold change in AMBRA1 sequencing results after Src knockdown, showing a statistically significant increase by 1.48‐fold (*p* < 0.05). Figure 6B confirms this at the protein level with western blot assay, revealing elevated AMBRA1 after Src knockdown. To investigate the role of PP1 in inhibiting TGF‐β1‐mediated EndMT, Figure 6C,D utilized a combination of TGF‐β1, PP1 and shRNA knockdown of AMBRA1. The results demonstrated that PP1's inhibition of TGF‐β1‐induced EndMT was reversed upon AMBRA1 knockdown. Similarly, Figure 6E indicates that solely knocking down AMBRA1 did not alter mitophagy, but PP1‐induced Src inhibition could activate mitophagy, which was diminished when AMBRA1 was knocked down, as evidenced by the decrease in LC3‐II and increase in TOMM20. This suggests PP1's Src inhibition‐induced mitophagy may depend on AMBRA1 activation. Since AMBRA1 activation can lead to mitophagy, involving mitochondrial ubiquitination, Figure 6F examined mitochondrial protein extraction and ubiquitination levels, using MG132. MG132 is used as a proteasome inhibitor to prevent the degradation of ubiquitinated proteins, allowing for the assessment of the ubiquitination levels of mitochondrial proteins. Without Src knockdown, no significant increase in mitochondrial ubiquitination was observed, whereas Src knockdown resulted in a notable increase at 12 and 24 h, implicating AMBRA1 in Src inhibition‐induced mitophagy. Parkin and PINK1 are part of a pathway that detects and marks damaged mitochondria for degradation through mitophagy. AMBRA1 is involved in autophagy initiation and may interact with Parkin to promote the clearance of damaged mitochondria. In Figure 6G, downstream pathways of AMBRA1, including parkin and pink1, were investigated, showing time‐dependent increases in both mitochondrial and total cellular contexts after Src knockdown. The densitometric quantification analysis is shown in Figure 6I. Binding of AMBRA1 to Parkin can facilitate the recruitment of Parkin to the mitochondria, enhancing its ubiquitin ligase activity. As shown in Figure 6H, showing co‐IF of AMBRA1 and Parkin, revealed increased expression and colocalization post‐Src knockdown, highlighting the significance of their interaction and Parkin's role in mitophagy through ubiquitination. Increased ubiquitination by Parkin signifies a heightened state of mitophagy, targeting damaged mitochondrial components for degradation and maintaining cellular homeostasis. Given that Parkin‐ubiquitylated mitochondria is necessary for combination between mitochondrion and autophagosome, whether ubiquitin level of Parkin was detected by immunocoprecipitation assay (co‐IP). Finally, in Figure 6J, co‐IP assessed Parkin ubiquitination levels, which were elevated after PP1‐mediated Src inhibition and reversed by AMBRA1 knockdown, while whole‐cell lysate results also showed a reversal of Parkin protein levels increase after AMBRA1 knockdown. For clarity of exposition, we further detected the expression of Parkin and LC3‐II with or without CQ which suppressed autophagy downstream degradation. By LC3‐turnover analyses using CQ we could demonstrate that PP1‐triggered LC3‐II expression was further elevated by CQ‐treatment and AMBRA1 knockdown alters this result. Furthermore, the inhibition of autophagy by CQ did not alter the levels of Parkin, indicating that the degradation of Parkin is not influenced by autophagy (Figure 6K).

There are three canonical pathways inducing mitophagy: ubiquitin‐dependent PINK1/Parkin and ubiquitin‐independent Bnip3L/Nix and Fundc1. However, when we examined the other two mitophagy pathways, there was no significant change in Bnip3 and Fundc1 protein levels after knocking down Src (Supplementary Figure 5A). It suggested that the Src is mostly excreted through the AMBRA1/Parkin. Here, we sought to determine whether, in addition to Parkin, Ambra1 affects mitophagy through other substrates. We predicted two potential substrates, RNF2 and MARCHF5, based on protein structure using Ubibrowser. Subsequent polymerase chain reaction (PCR)c analysis showed no structural changes, suggesting that Parkin might play a more predominant role (Supplementary Figure 5B,C). In the rescue experiment, we found that the increase of PP1‐activated Ambra1 was not affected after Parkin was knocked down (Supplementary Figure 5D), so it was still suggested that Parkin was downstream of Ambra1. Although parkin acts as an E3 ubiquitin ligase, it does not affect the expression of Ambra1, but uses mitochondrial outer/inner membrane protein as the substrate for ubiquitination. Finally, to assess the affinity of AMBRA1 towards its target Parkin, we conducted a molecular docking analysis. The 3D coordinates of the two proteins were downloaded from the Protein Data Bank at http://www.rcsb.org/. Using Autodock Vina v.1.2.2, we obtained the binding poses and interactions between the two proteins, generating the binding energies for each interaction. The results showed that the two proteins bind through visible hydrogen bonds and strong electrostatic interactions. Furthermore, the two candidates, AMBRA1 and Parkin, have a binding energy of −4.464 kcal/mol (Supplementary Figure 5E). Generally, a binding energy lower than −4.25 kcal/mol indicates a certain binding activity between a ligand small molecule and a receptor protein, suggesting a relatively stable binding between the two.

### 3.7 Mitophagy inhibition‐induced EndMT depended on IL‐6 secretion

**FIGURE 7:**
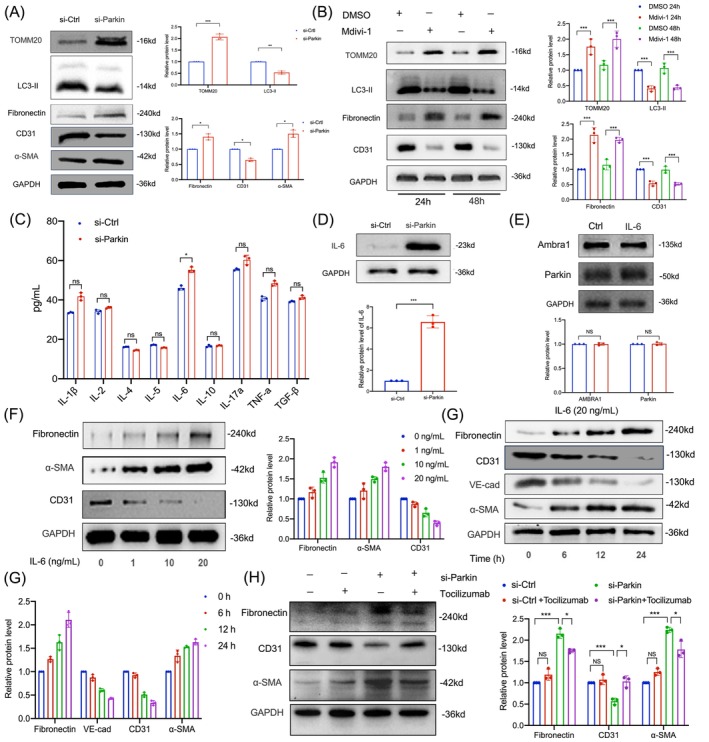
Mitophagy inhibition‐induced EndMT depended on IL‐6 secretion. HUVECs were transfected with si‐Ctrl or si‐Parkin for 24 h. (A) Western blot analysis and densitometric quantification of LC3‐II, TOMM20, fibronectin, CD31 and α‐SMA expression in si‐Ctrl or si‐Parkin groups (*n* = 3). (B) HUVECs treated with 20 μM mdivi‐1 and 20 μM DMSO for 24 h. Western blot analysis and densitometric quantification of LC3‐II, TOMM20, fibronectin, CD31 and α‐SMA expression in different groups (*n* = 3). (C) Inflammatory cytokines were measured in the cell supernatants by ELISA in si‐Ctrl or si‐Parkin groups (*n* = 3). (D) Western blot analysis and densitometric quantification of IL‐6 expression in si‐Ctrl or si‐Parkin groups (*n* = 3). (E) HUVECs were treated with IL‐6 (20 ng/mL) for 24 h. Western blot analysis and densitometric quantification of AMBRA1 and Parkin expression in different groups (*n* = 3). (F) HUVECs were treated with different concentration IL‐6 for 24 h. Western blot analysis and densitometric quantification of fibronectin, CD31 and α‐SMA expression in different groups (*n* = 3). (G) HUVECs were treated with IL‐6 (20 ng/mL) for different times. Western blot analysis and densitometric quantification of fibronectin, CD31 and α‐SMA expression in different groups (*n* = 3). (H) HUVECs were treated with si‐Ctrl, si‐Src and/or *tocilizumab* (50 μg/mL) for 24 h. Western blot analysis and densitometric quantification of fibronectin, CD31 and α‐SMA expression in different groups (*n* = 3). Data were presented as mean ± SEM. *p < 0.05; ***p < 0.001. ELISA, enzyme‐linked immunosorbent assay; EndMT, endothelial‐to‐mesenchymal transition; HUVECs, human umbilical vein endothelial cells; IL‐6, Interleukin‐6; α‐SMA, α‐smooth muscle actin.

To examine the impact of mitophagy on EndMT, we conducted experiments in HUVECs by knocking down Parkin via siRNA or by treating the cells with mdivi‐1 to inhibit autophagy. Post‐transfection, we observed a decrease in LC3‐II, an increase in TOMM20 expression, with downregulation of CD31 and upregulation of α‐SMA and FN (Figure 7A). Mdivi‐1 treatment yielded similar autophagy‐blocking effects in HUVECs (Figure 7B), suggesting that inhibition of autophagy directly promotes EndMT.

Various inflammatory cytokines may influence autophagic flux. We used enzyme‐linked immunosorbent assay (ELISA) to detect changes in secreted substances from HUVECs after Parkin knockdown, including an array of cytokines. The concentration of IL‐6 was notably higher in HUVECs transfected with Parkin siRNA compared to control siRNA (Figure 7C). To verify if IL‐6 expression in endothelial cells corresponded with its concentration in the cell supernatant, we performed a western blot analysis and observed a significant increase in IL‐6 at the protein level in the si‐Parkin group (Figure 7D).

Exploring the potential mechanisms behind IL‐6's promotion of EndMT, we stimulated HUVECs with recombinant human IL‐6. Recovery experiments post‐IL‐6 intervention revealed no feedback loop affecting Parkin and AMBRA1 (Figure 7E). To determine the optimal in vitro concentration of IL‐6, we treated cells with various concentrations (0, 1, 10 and 20 ng/mL) for 24 h and analysed the EndMT markers via western blot assay. At 20 ng/mL IL‐6 for 24 h, there was a significant decrease in CD31 and an increase in α‐SMA and FN (Figure 7F,G), with a time‐dependent induction of EndMT in HUVECs (Figure 7F,G). Tocilizumab is an IL‐6R neutralizing antibody that blocks IL‐6 from binding to IL‐6R. Following si‐Parkin to inhibit mitophagy, we used Tocilizumab to investigate the relationship between Parkin and IL‐6. Western blot assay results indicated that EndMT levels were elevated after Parkin knockdown. However, when Tocilizumab was used to counteract the production of IL‐6, the increased trend of EndMT was reversed (Figure 7H). These findings suggest that mitophagy inhibition induces EndMT by increasing IL‐6 secretion.

### 3.8 AMBRA1/Parkin‐mediated mitophagy in transplanted kidney rejection and CAD patients

**FIGURE 8:**
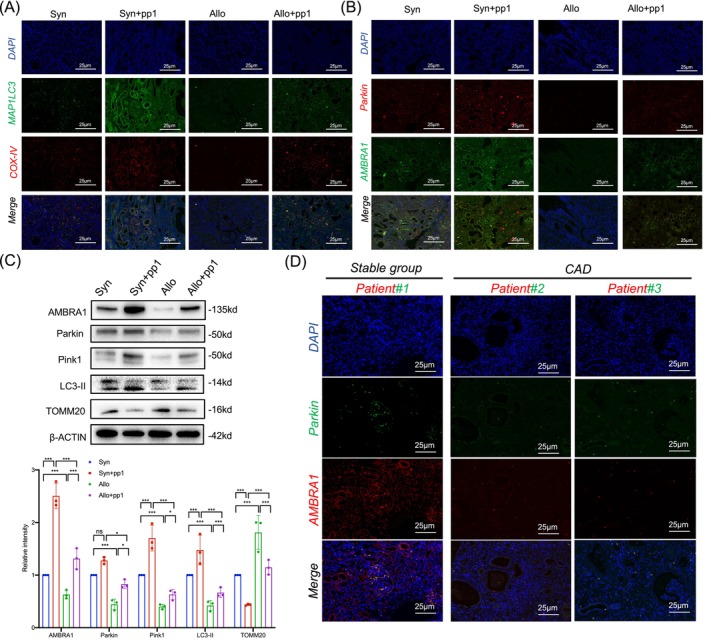
AMBRA1/Parkin‐mediated mitophagy in transplanted kidney rejection and CAD patients. (A) Representative immunofluorescence staining images of COX‐IV and MAP1LC3 expression in transplanted kidney tissues from the four groups (*n* = 6) (bar = 25 μm). (B) Representative immunofluorescence staining images of COX‐IV and MAP1LC3 expression in transplanted kidney tissues from the four groups (*n* = 6) (bar = 25 μm). (C) Western blot analysis and densitometric quantification of AMBRA1, Parkin, Pink1, IL3‐II and TOMM20 expression in transplanted rat kidney tissues from the four groups (*n* = 6). (D) Representative immunofluorescence staining images of AMBRA1 and Parkin expression in transplanted kidney tissues in the stable group and CAD group (*n* = 5) (bar = 25 μm). Data were presented as mean ± SEM. *p < 0.05; ***p < 0.001. CAD, chronic allograft dysfunction.

In a chronic allogeneic transplanted kidney rejection rat model, we investigated the effect of PP1‐mediated Src inhibition on mitophagy. IF assay revealed a significant decrease in MAP1LC3 expression and its colocalization with the mitochondrial marker COX‐IV in the Allo group compared to the Syn group. However, the PP1 + Allo group showed increased renal tissue LC3 expression and more colocalization with COX‐IV than the Allo group (Figure 8A). Additionally, immunofluorescent double staining for Parkin and AMBRA1 across four rat groups showed that compared to the Syn group, the expression and colocalization of Parkin and AMBRA1 were significantly reduced in the Allo group but were reversed with PP1 treatment (Figure 8B). To further confirm the in vivo changes in mitophagy after altering Src activity, protein immunoblotting of related markers corroborated the fluorescence findings. The Allo group had decreased levels of LC3‐II, Parkin, Pink1 and AMBRA1, and increased TOMM20, suggesting reduced mitophagy post‐transplant of kidney, which was reversed by PP1 intervention (Figure 8C). Similarly, IF staining in human subjects for Parkin and AMBRA1 expression and colocalization found reduced levels of them in CAD patients, further confirming that AMBRA1/Parkin‐mediated mitophagy is inhibited after kidney transplantation (Figure 8D).

## 4 DISCUSSION

In this study, we conducted both in vivo and in vitro experiments to elucidate the interplay between mitophagy and the process of EndMT in CAD patients. Our findings can be summarized as follows: (1) Sc‐RNA seq analysis showed that Src pathway activated and related to the program of EndMT in CAD recipients; (2) Src suppression ameliorated EndMT and renal allograft fibrosis by activating AMBRA1/Parkin‐induced mitophagy; (3) mitophagy blockage promoted EndMT and renal allograft fibrosis in an IL‐6‐dependent way. These results clearly demonstrated the protective effect of mitophagy on EndMT and the latent treatment of Src suppression on renal fibrosis after kidney transplantation.

Src was originally identified as a proto‐oncogene, and genetic mutations that result in increased activity or its over‐expression are frequently found in human tumours from the colon, liver, lung, breast and pancreas.^23^ Src kinase activation plays a pivotal role in the pathogenesis of organ fibrosis, a process marked by chronic inflammation and maladaptive repair that significantly impairs organ function and patient quality of life.^24^ To date, Src kinase family is worth investigating as a potential organ fibrosis clinical treatment target. However, the current literature of the anti‐fibrotic effect of Src is lacking in interstitial fibrosis of transplanted kidney. In this study, we identified the role of Src inhibitor PP1 on renal vascular EndMT and allograft fibrosis after kidney transplantation. Our study fills this gap in the field.

In our previous research, dynamic changes in autophagy flux were closely associated with the process of interstitial fibrosis in transplanted kidneys.^29^ Clinical experiments have shown that combination therapy with the Src inhibitor Saracatinib reduced the growth of xenografts in ovarian cancer by enhancing autophagy.^24^ Additionally, our next‐generation sequencing results also indicated that treatment of endothelial cells with the PP1 similarly activated mitophagy/autophagy‐related pathways. Beneficial effect of mitophagy on EndMT has been recognized in our study. This is consistent with our previous finding of reduced ATG16L‐mediated autophagic flux in late kidney transplantation.^29^ According to our analysis, mitophagy knockdown promoted the progression of renal vascular EndMT and allograft fibrosis, showing antifibrotic role of mitophagy in kidney transplantation. Takagaki et al. found that disruption of endothelial autophagy could result in significant pathological EndMT and kidney fibrosis using endothelial‐specific Atg5 knockout mice (Atg5 Endo; *Cdh5‐Cre Atg5* ^*flox/flox*^ mice).^20^ In our rat renal allogenic transplant model, significantly decreased mitophagy was observed in the Allo group compared with the Syn group. Furthermore, inhibition of Src with PP1 enhanced mitophagy‐related proteins and attenuated fibrosis‐related proteins, in line with previous studies on Src inhibition.^24^ In addition, we further explored underlying mechanisms of interaction on AMBRA1 and Parkin. Parkin, an E3 ubiquitin ligase, is recruited to the surface of damaged mitochondria, where it ubiquitinates various mitochondrial proteins to mark them for degradation.^30^ AMBRA1, a key regulator in autophagy, interacts with the Beclin 1 complex to promote autophagosome formation. Previous studies have only shown that AMBRA1 may combine Parkin and help identify damaged mitochondria in mitophagy.^31^ The specific mechanism needs to be further elaborated. Here, we elucidate that AMBRA1 enhances mitochondrial ubiquitination and mitophagy by stabilizing Parkin's ubiquitination levels and influencing its translocation to mitochondria. Thus, our study findings provide new evidences of protective role of mitophagy and therapeutic effect of Src/AMBRA1/Parkin pathway on renal allograft fibrosis.

To detect the cytokines alteration after impaired mitophagy, supernatant of HUVECs were measured by ELISA and we identified that IL‐6 increased significantly and was related to the development of EndMT. Application of IL‐6 neutralizing antibodies could reverse Parkin siRNA‐induced EndMT. In kidney transplantation, critical roles of IL‐6 have been identified in innate immune responses and adaptive immunity. IL‐6 can mediate cell‐mediated rejection, ABMR and chronic allograft vasculopathy.^32^ Emerging data showed that treatment with anti‐IL‐6 antibody could restrain the activity and progression of late ABMR.^33^ Moreover, IL‐6 increases the expression of fibroblast growth factor 23, which then contributes to excessive accumulation of extracellular matrix, resulting in renal interstitial fibrosis and CAD.^34^ However, the mechanism of IL‐6‐induced EndMT in kidney transplantation was not explored in detailed in our study. Therefore, we intend to elaborate the molecular mechanism in the following studies.

In our study, defective mitophagy increased IL‐6 production in HUVECs, whereas no change in mitophagy‐related proteins such AMBRA1 and Parkin was observed under the condition of incubation of IL‐6 and endothelial cells. Similarly, Takagaki et al. reported inhibition of autophagy in endothelial cells resulted in the synthesis and secretion of IL‐6.^20^ Autophagy plays multiple roles in inflammatory responses^35^ and IL‐6 is a critical cytokine involved in the pathogenesis of inflammation.^36^ Peng et al. found that deficient ATG5 in proximal tubular epithelial cells could increase IL‐6 production and enhance nuclear translocation of p65 and transcriptional activity of NF‐κB,^37^ suggesting that autophagy deficiency may induce IL‐6 secretion by NF‐κB signalling pathway activation. Meanwhile, decreased ATG5 expression in salivary gland epithelial cells contributed to increased IL‐6 by activating JAK–STAT pathway.^38^ Meanwhile, Autophagy is a catabolic process in response to diverse conditions of stress including oxidative stress.^39^ Increased ROS and altered mitochondrial dynamics can accelerate autophagy/mitophagy. In our study, silencing of Src in endothelial cells leaded to a higher mitoSOX level, indicating increased production of ROS. These data suggested ROS played a critical role in IL‐6 synthesis and secretion, consistent with Di Luigi's reports.^40^ Hence, the relationship between mitophagy and IL‐6 production is complicated and may be attributed to a variety of physiological process.

In conclusion, our study demonstrated that blocking activation of Src kinase by PP1 could alleviate renal allograft fibrosis in rats presumably by AMBRA1/Parkin‐induced mitophagy activation. EndMT induced by mitophagy inhibition is dependent on IL‐6 production. These provide the underlying therapeutic strategies of targeting mitophagy for preventing renal allograft fibrosis.

## AUTHOR CONTRIBUTIONS

Min Gu and Ruoyun Tan designed the study. Dengyuan Feng, Zeping Gui, Zhen Xu, Xiaobin Ju and Zhou Hang performed the experiments. Dengyuan Feng, Zeping Gui, Li Sun, Shuang Fei, Hao Chen, Jun Tao and Ruoyun Tan acquired and analysed the data. Dengyuan Feng and Ruoyun Tan wrote the paper. Zijie Wang and Min Gu revised the manuscript. All authors read and approved the final manuscript.

## FUNDING INFORMATION

This work was supported by the National Natural Science Foundation of China (grant numbers 82270790, 82170769, 82070769, 81900684, 81870512, 81770751), Jiangsu Province Natural Science Foundation Program (grant number BK20191063), “333 High Level Talents Project” in Jiangsu Province (grant numbers BRA2017532, BRA2016514), Xuzhou Medical University Outstanding Talents Fund Project (grant numbers XYFY202227), Huaian First People's Hospital High‐level Talent Project (grant numbers GQ202208), Postgraduate Research & Practice Innovation Program of Jiangsu Province (grant numbers KYCX22_1776).

## CONFLICT OF INTEREST STATEMENT

The authors declare no conflicts of interest.

## CONSENT TO PARTICIPATE

All recipients signed informed consent forms for the permission to use their tissues for scientific purpose. The procedures in our study follow the ethical standards of the Declarations of Helsinki and Istanbul.

## CONSENT FOR PUBLICATION

I understand that the information will be published without my/my child or wards/my relatives, but that full anonymity cannot be guaranteed. I understand that the text and any pictures or videos published in the article will be freely available on the Internet and may be seen by the general public. The pictures, videos and text may also appear on other websites or in print, may be translated into other languages or used for commercial purposes. I have been offered the opportunity to read the manuscript.

## Supporting information


**Data S1.** Supporting Information.

## Data Availability

The data that support the findings of this study are available in Gene Expression Omnibus (GEO) at https://www.ncbi.nlm.nih.gov/geo/query/acc.cgi?acc=gse195718. These data were derived from the following resources available in the public domain: Frontiers in Transplantation, https://doi.org/10.3389/frtra.2022.988238.
