# Supplementary material for: Src inhibition modulates AMBRA1‐mediated mitophagy to counteract endothelial‐to‐mesenchymal transition in renal allograft fibrosis
Source: Cell Prolif. 2024 Jun 29;57(11):e13699. doi: 10.1111/cpr.13699 (PMC11533082; doi:10.1111/cpr.13699)
Supplement: Supplementary file 1 — Data S1. Supporting Information. [file CPR-57-e13699-s001.zip › cpr_13699_supp info_captions.docx]

**Src Inhibition Modulates AMBRA1-Mediated Mitophagy to Counteract Endothelial-to-Mesenchymal Transition in Renal Allograft Fibrosis**

**Supplementary Figure 1**

**A:** Single-cell sequencing data quality control. **B:** Single-cell RNA-sequencing results identify cell types that express Src.

**Supplementary Figure 2**

**A**: Representative images of IHC staining and Statistical graphs of p-Src expression in renal allograft tissues in the stable group antibody-mediated rejection (ABMR) and T-cell mediated rejection (TCMR) (n=6) (Bar=25μm). Data were presented as mean ± SEM. NS, non-significant.

**Supplementary Figure 3**

**A**: HUVECs were treated with 10ng/ml TGF-β1 for 0-48 hours. Western blot analysis (A) and densitometric quantification (C) of p-Src, fibronectin, CD31 and α-SMA expression (n=3). **B**: HUVECs were treated with different concentration TGF-β1 for 24 hours. Western blot analysis (B) and densitometric quantification (C) of p-Src, fibronectin, CD31 and α-SMA expression (n=3). **D**: The live HRGECs were photographed (Bar = 10，20μm) . **E**: HRGECs were treated with 10ng/ml TGF-β1 for 0-48 hours. Western blot analysis (E) and densitometric quantification (G) of p-Src, fibronectin, CD31 and α-SMA expression (n=3). **F**: HRGECs were treated with different concentration TGF-β1 for 24 hours. Western blot analysis (F) and densitometric quantification (G) of p-Src, fibronectin, CD31 and α-SMA expression (n=3). **H-I**: HRGECs were treated with 20ng/ml TGF-β1 and different concentration PP1 for 24 hours. Western blot analysis (H) and densitometric quantification (I) of p-Src, fibronectin, CD31 and α-SMA expression (n=3). Data were presented as mean ± SEM. *p < 0.05, **p < 0.01, ***p < 0.001.

**Supplementary Figure 4**

**A**: KEGG pathway network diagram

**Supplementary Figure 5**

**A**: HUVECs were transfected with si-Ctrl or si-Src for 0 and 12 hours. After transfection, western blot analysis and densitometric quantification of BNIP3 and FUNDC1 expression (n=3). **B**: UbiBrowser showed substrate proteins that might interact with AMBRA1. **C**: RNF2 and MARCHF5 *RNA* was analyzed by real-time *PCR*. **D**: HUVECs were transfected with si-Ctrl or si-Parkin for 24 hours. After transfection, cells were treated with 20ng/ml PP1 for 24 hours. Western blot analysis and densitometric quantification of AMBRA1 expression (n=3). **E**: Binding mode of protein to their targets by molecular docking. Binding mode of AMBRA1 to Parkin. Data were presented as mean ± SEM. *p < 0.05, **p < 0.01, ***p < 0.001.
