# Supplementary figures and images for: Src inhibition modulates AMBRA1‐mediated mitophagy to counteract endothelial‐to‐mesenchymal transition in renal allograft fibrosis
Source: Cell Prolif. 2024 Jun 29;57(11):e13699. doi: 10.1111/cpr.13699 (PMC11533082; doi:10.1111/cpr.13699)

(A)

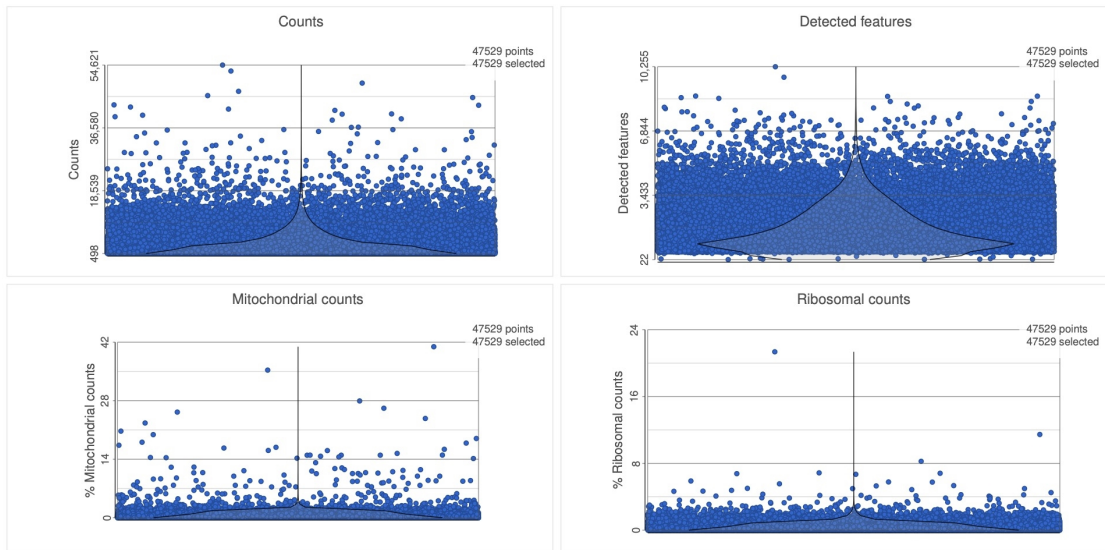

(B)

***P* Value : CAD VS. Stable**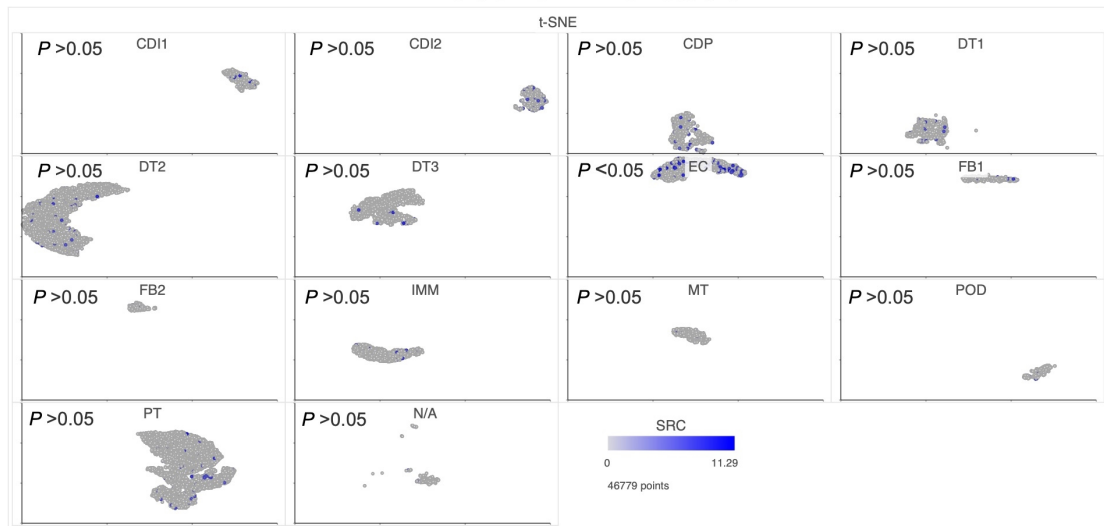

Supplement: Supplementary file 1 — Data S1. Supporting Information. [file CPR-57-e13699-s001.zip › Supplementary Figure1.pdf]

(A)

*Stable group**ABMR**TCMR**p-Src*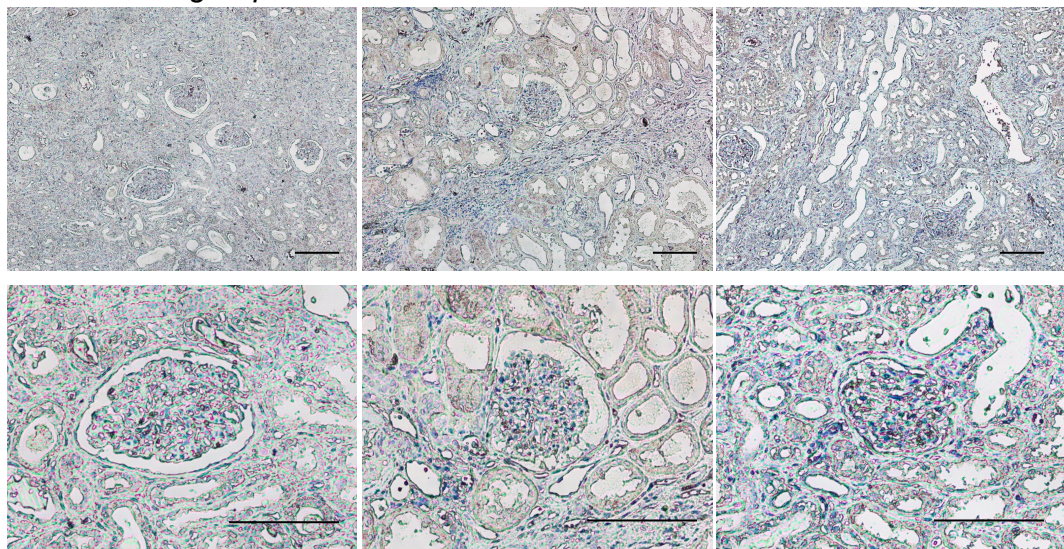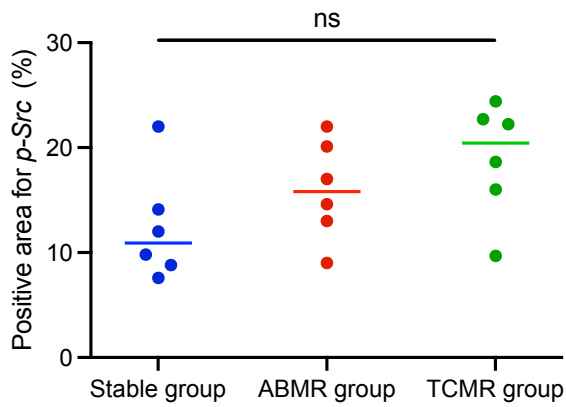

Supplement: Supplementary file 1 — Data S1. Supporting Information. [file CPR-57-e13699-s001.zip › Supplementary Figure2.pdf]

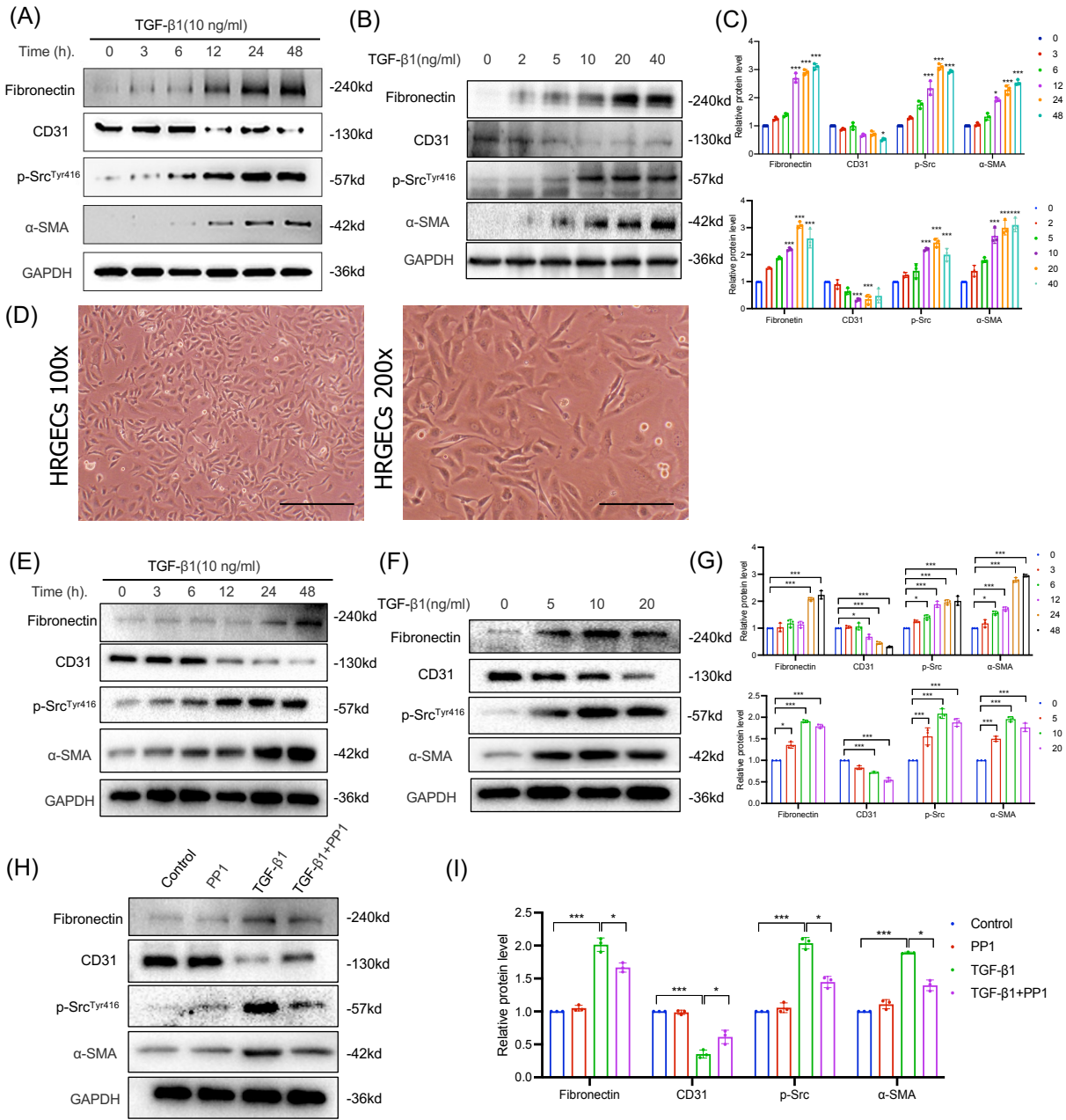

Supplement: Supplementary file 1 — Data S1. Supporting Information. [file CPR-57-e13699-s001.zip › Supplementary Figure3.pdf]

# MITOPHAGY - ANIMAL

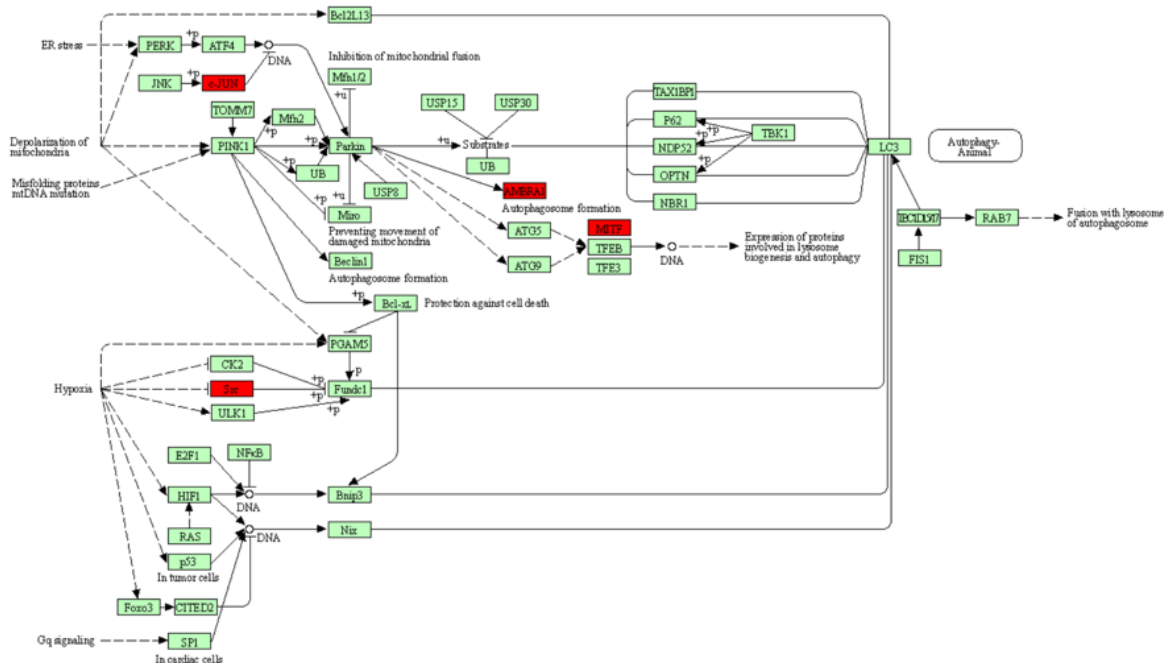

Supplement: Supplementary file 1 — Data S1. Supporting Information. [file CPR-57-e13699-s001.zip › Supplementary Figure4.pdf]

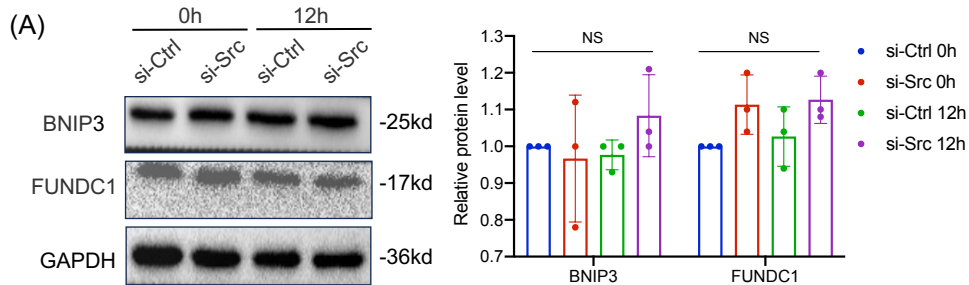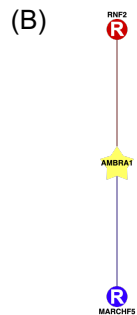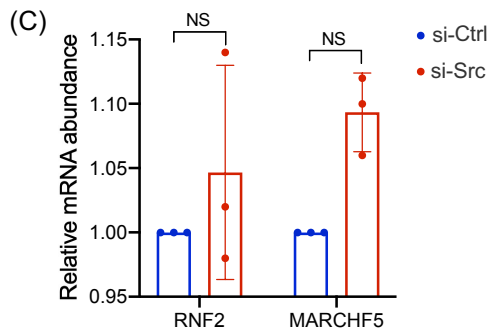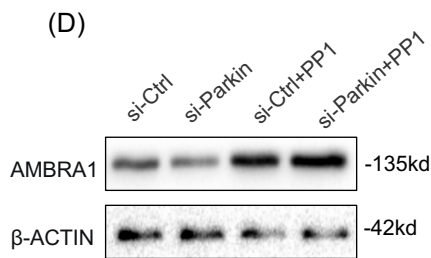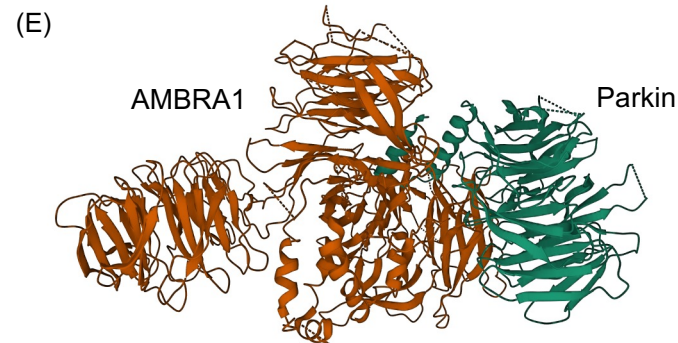

Supplement: Supplementary file 1 — Data S1. Supporting Information. [file CPR-57-e13699-s001.zip › Supplementary Figure5.pdf]
